# Field assessment of 4-hydroxycoumarin as an attractant for anthropophilic *Anopheles* spp. vectors of malaria in Madagascar

**DOI:** 10.1038/s41598-020-59822-6

**Published:** 2020-02-20

**Authors:** Tovo Mbolatiana Andrianjafy, Voahangy Vestalys Ramanandraibe, Elodie Toavina Andrianarijaona, Niry Hasinandrianina Ramarosandratana, Lala Harivelo Ravaomanarivo, Patrick Mavingui, Marc Lemaire

**Affiliations:** 1 International Associated Laboratory, University of Antananarivo-Lyon 1, PO Box 906, Antananarivo, Madagascar; 2 Department of Entomology, University of Antananarivo, PO Box 906, Antananarivo, Madagascar; 3 University of La Réunion, UMR PIMIT, INSERM 1187, CNRS 9192, IRD 249, Plateforme CYROI, 2 Rue Maxime Rivière, Sainte Clotilde, La Réunion France; 4 ICBMS, CNRS, UMR 5246, University of Claude Bernard Lyon, 1 rue Victor Grignard, Bâtiment Lederer, 69622 Villeurbanne Cedex, France

**Keywords:** Behavioural ecology, Malaria

## Abstract

Mosquito-borne diseases like malaria are a major public health problem in tropical countries, such as Madagascar. Female *Anopheles* mosquito vectors the human malaria parasites (*Plasmodium* spp.) and is important indicator in malaria surveillance activities. Among the various means of vector control in Madagascar, the use of attractants for mass trapping of target species could be an alternative to insecticides. The aim of this study is to evaluate whether 4-hydroxycoumarin can be used as an attractant for anthropophilic *Anopheles* spp. vectors of malaria. For this, a field study was conducted using CDC light traps in the village of Ambohidray, Madagascar. 16 days of trapping was conducted and four replicates nights were performed for each product tested. 4-hydroxycoumarin, octenol and two types of blend of these products were tested. The results showed that 4-hydroxycoumarin (2 mg) have a significant attractive effect on *Anopheles* spp. and significant selectivity towards *Anopheles gambiae s.l*, and *Anopheles mascarensis* which are both significant malaria vectors in Madagascar. A synergy of 4-hydroxycoumarin with octenol was found to attract these mosquito vectors. A significant decrease in vector populations was observed during this experiment. These results suggest that 4-hydroxycoumarin could be useful for malaria surveillance and the control of vector populations.

## Introduction

Mosquito-borne diseases present a major public health threat in tropical and subtropical areas and disproportionately impact developing nations, such as Madagascar^1^. Among numerous mosquito vectors existent in Madagascar several *Anopheles* species are responsible for the transmission of malaria parasites^2–4^. Due to their distribution, malaria is endemic to 90% of the country^5^ and is the fourth leading cause of death in Madagascar^6^.

In order to limit the transmission of malaria, several methods of control have been implemented such as the use of long lasting insecticide-treated mosquito nets (LLINs)^7^, and indoor residual spraying (IRS)^8^ to protect populations in regions with a high potential for epidemics^9^. These control methods have shown significant efficacy, but they are also insufficient in the long term due to the rapid amplification of insecticide resistance in vector populations^10,11^. In addition, controlling mosquito populations through the use of larvicides and adulticides has found to have negative impacts on ecosystems^12–14^. For these reasons, the explorations of other more sustainable and environmentally friendly alternatives are encouraged worldwide^15,16^.

The use of attractants for selective and mass trapping has been suggested as a promising complementary to insecticide for malaria vector surveillance, control and reduction^17–19^. A recent study has shown that 4-hydroxycoumarin, a compound derived from coumarin, a natural substance of plant origin, has a significant attractiveness activity on the mosquito *Aedes albopictus*, a mosquito vector of Dengue and Chikungunya, in laboratory and field assays^20^. Preliminary field tests in the east of Madagascar, using CDC light traps, have shown that the same compound also attracts *Anopheles* mosquito species. Among the caught species, primary malaria vectors such as *Anopheles gambiae* complex, *Anopheles mascarensis* and *Anopheles funestus* were captured^21^. However, the results obtained were not sufficient to confirm this attractive property of 4-hydroxycoumarin toward *Anopheles*. Thus, a new field investigation was carried out for the evaluation of the attractiveness of this compound to the following anthropophilic malaria vectors: *An. gambiae s.s*, *An. mascarensis*, *An. funestus* in Madagascar.

## Results

**Figure 1:**
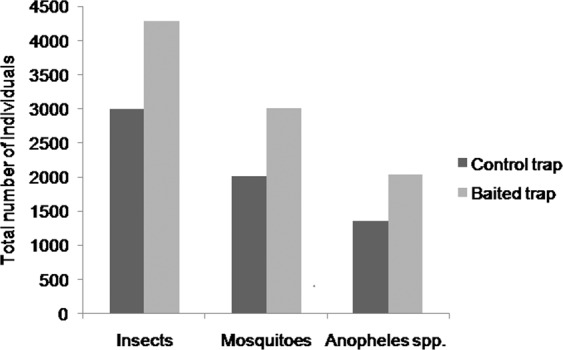
Total number of insects, mosquitoes and *Anopheles* spp. caught in control and baited traps in the village of Ambohidray.

A total of 7265 insects were collected from the Ambohidray site, of which 2989 and 4276 were captured in control and baited traps respectively. These insects were represented by five orders, including Diptera, Lepidoptera, Coleoptera, Hymenoptera and Hemiptera. Among the Diptera, 5010 (69%) were mosquitoes belonging to the family Culicidae, of which 2002 (40%) were captured in control traps and 3008 (60%) in baited traps. In this family, two genera: *Culex* and *Anopheles* were identified. The total number of *Anopheles* collected was 3384 (68%) of which 1348 (39.8%) in the control traps and 2036 (60.2%) in the baited traps (Fig. 1).

Statistically, no significant differences were observed between the mean numbers of insects, mosquitoes and *Anopheles* spp. in the baited trap and those captured in the control trap (*P* > *0.05*) for all products tested. For *Culex*, three species were caught including *Cx. decens*, *Cx. tritaeniorynchus* and *Cx. giganteus*. In the case of *Anopheles*, three species were identified morphologically: *An. squamosus*, *An. coustani*, *An. mascarensis* and the *An. gambiae* complex or *An. gambiae s.l* was also captured, but not identified at species level. The total numbers of *Anopheles gambiae s.l*, and *Anopheles* species collected in the control and baited traps are shown in (Table 1).

**Table 1:** Total number of *Anopheles gambiae s.l* and *Anopheles* species and captured in control and baited traps.

Products	*An. gambiae s.l*		*An. mascarensis*		*An. coustani*		*An. squamosus*	
	Control	Baited	Control	Baited	Control	Baited	Control	Baited
4-hydroxycoumarin (2 mg)	31	142	1	40	429	309	263	197
Octenol (2 mg)	52	80	0	0	334	526	28	97
4-hydroxycoumarin (10 mg)	21	60	0	0	87	413	24	42
4-hydroxycoumarin (10 mg) + octenol (2 mg)	3	17	0	2	27	43	22	25
4-hydroxycoumarin (10 mg) + octenol (2 mg) + CO_2_	3	6	0	0	14	26	9	11

### Average number of mosquitoes and *Anopheles* spp. in baited traps

The average number of mosquitoes in baited traps was significantly different (*F* = *8.44, P* < *0.001*) for tested products. We observed two phenomena simultaneously: a decrease in insect captures that was associated with the duration of trapping. More *Anopheles* malaria vectors were captured in the baited traps compared to the control in all cases. The *LSD* multiple comparison test showed that traps baited with 4-hydroxycoumarin (2 mg) within the first trapping days and octenol (2 mg) in the second trapping period had the highest mean number of mosquitoes captured, 213 and 234 individuals, respectively. They were followed by 4-hydroxycoumarin (10 mg) with an average of 152 individuals during the third trapping period. The lowest number of captured mosquitoes was observed during the fourth and the fifth trapping period in the presence of the product combinations: 4-hydroxycoumarin (10 mg) plus octenol (2 mg) and 4-hydroxycoumarin (10 mg) plus octenol (2 mg) plus CO_2_. There were less than 30 individual mosquitoes captured with these blends; however, these tests were performed at the end of the field experiment.

Significant differences were also observed for the average number of *Anopheles* spp. including anthropophilic and zoophilic species in the baited traps of the different products (*F* = *7.52, P* < *0.001*). *LSD* analysis showed that the traps with octenol (2 mg) had the highest average number of *Anopheles* spp. with 175 individuals, followed by 4-hydroxycoumarin with a mass of 2 mg and 10 mg with 114 and 128 individuals, respectively. As in the previous results, the mean number of *Anopheles* spp. was lower in the two combinations: 4-hydroxycoumarin (10 mg) plus octenol (2 mg) and 4-hydroxycoumarin (10 mg) plus octenol (2 mg) plus CO_2_ with only 10 and 20 individuals, respectively.

### Comparison of average number of mosquitoes in control and baited traps

The effect of the tested products on the number of *Anopheles* vectors captured was examined and significant differences were observed between the number of the two malaria vectors together: *An. gambiae s.l* plus *An. mascarensis* in the control and baited traps for the first test (*P* < *0.001*) for 4-hydroxycoumarin (2 mg). The mean number of *An. gambiae s.l* plus *An. mascarensis* in the baited trap was significantly higher with 30 individuals than that recorded in the control trap with 5 individuals. Similar results were also observed in the presence of 4-hydroxycoumarin (10 mg) in combination with octenol (2 mg) (*P* < *0.001*) with a mean number of 5 individuals captured in the baited trap and 0 individual in the control trap. In the case of the other compounds, analysis showed no significant difference between the mean number of malaria vectors in the control and baited traps specifically because their number became too small.

**Figure 2:**
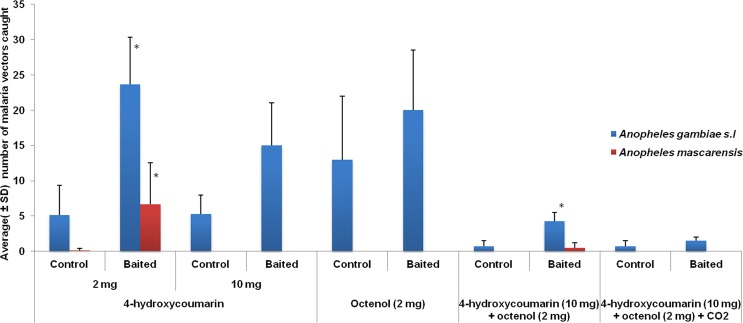
Average (±SD) of the number of *An. gambiae s.l*, and *An. mascarensis* captured in control and baited traps. Asterix in the graph denotes that the mean number of individuals in baited traps is significantly higher than that in control traps at *P* < *0.05* based on *t-test* analysis.

For each *Anopheles* species, results showed that there were significant differences between the mean number of *An. gambiae s.l* in both traps (*P* < *0.001*) for 4-hydroxycoumarin (2 mg). The mean number of *An. gambiae s.l* in the baited trap was significantly higher with 24 individuals than that recorded in the control with 5 individuals. Similar results were also observed for the species *An. mascarensis* (*P* = *0.04*) with the same compound using the same amount of 2 mg with a mean number of 7 individuals recorded in the baited trap and 0 individual in the control trap. It was the same in the case of *An. gambiae s.l* in the presence of the 4-hydroxycoumarin (10 mg) plus octenol (2 mg) combination (*P* = *0.012*) (Fig. 2). For *An. coustani* and *An. squamosus*, statistically there was no significant difference between the mean number of mosquito individuals in the control and baited traps for all tested products.

Comparing the mean number of individuals caught in the baited traps for each *Anopheles* species, analysis showed that statistically there were significant differences between the mean number of *An. gambiae s.l* for the different tested products (*F* = *18.48, P* < *0.0001*). The highest mean was recorded in the first test using 4-hydroxycoumarin (2 mg) with 24 individuals. It was lower for blend 4-hydroxycoumarin (10 mg), octenol (2 mg) and CO_2_ with only 2 individuals. Similar results were also observed for *An. mascarensis* (*F* = *3.75, P* = *0.01*) and *An. squamosus* (*F* = *9.34, P* < *0.001*) with respectively 7 and 33 individuals on average for 4-hydroxycoumarin (2 mg). In the case of *An. coustani*, significant difference was also observed between the mean number of individuals for the different tested products (*F* = *4.71, P* = *0.007*) but the highest average was recorded for octenol (2 mg) with 131 individuals.

### Kairomone index

For 4-hydroxycoumarin (2 mg), the kairomone index was almost negligible for mosquitoes. It was less than 7%. This was especially observed for *Anopheles* spp. However, the KI was higher for the malaria vectors: *An. gambiae s.l* plus *An. mascarensis* with 70%. For the same product but with a mass of 10 mg, KI for mosquitoes and *Anopheles* spp. were close to 50%. For malaria vectors, KI was 48%. Similar results were observed in the second test, octenol (2 mg), but the KI for mosquitoes, *Anopheles* spp. and malaria vectors ranged from 20% to 28% indicating no selectivity for malaria vector species. For the blend 4-hydroxycoumarin (10 mg) and octenol (2 mg), the KI for malaria vectors was higher with 72%, although of that *Anopheles* spp. was 25%. For mosquitoes, KI was very low with only 9%. The KI for mosquitoes was very low also in the case of the blend 4-hydroxycoumarin (10 mg), octenol (2 mg) and CO_2_. In this combination, KI of *Anopheles* spp. and malaria vectors were 24% and 33%, respectively.

**Figure 3:**
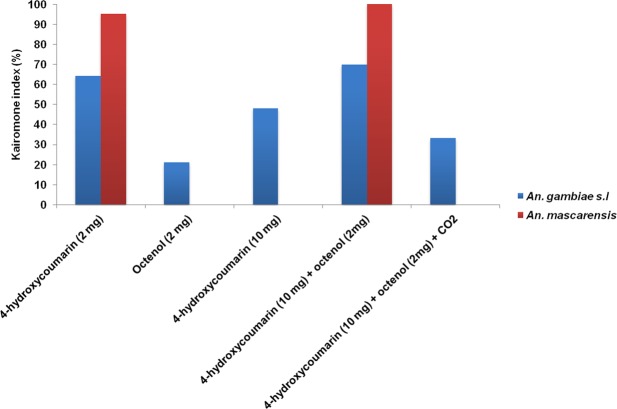
Kairomone indexes (%) of *An. gambiae s.l*, and *An. mascarensis* for all tested products.

For *An. gambiae s.l*, kairomone indexes were higher for 4-hydroxycoumarin (2 mg) and the blend 4-hydroxycoumarin (10 mg) and octenol (2 mg) with 64% and 70%, respectively. For 4-hydroxycoumarin (10 mg), KI of this *An. gambiae* complex was 48%. KI were low for octenol (2 mg) and the blend 4-hydroxycoumarin (10 mg), octenol (2 mg) and CO_2_ with less than 33%. For *An. mascarensis*, Kairomones indexes were very higher for 4-hydroxycoumarin (2 mg) and the blend 4-hydroxycoumarin (10 mg) and octenol (2 mg) with 95% and 100%, respectively. In contrast, KI of *An. mascarensis* were zero for the other products (Fig. 3).

**Figure 4:**
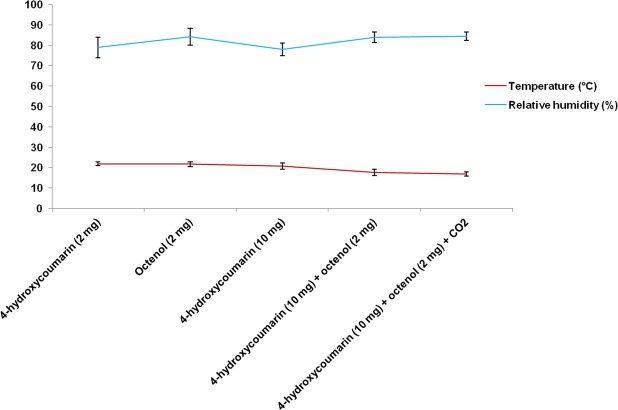
Averages (±SD) Temperature (°C) and relative humidity (%) recorded during the sampling periods.

On average, the temperature recorded at the site during this study was about 19.8 °C and the relative humidity was around 81.8%. Statistically, no significant differences were observed between the averages of relative humidity during this experiment. A similar result was also found for the averages of temperature. However, temperatures observed in the last two sampling periods appeared to be lower than the others around 17 °C (Fig. 4).

## Discussion

Our results showed that in the presence of both light and kairomone the number of Diptera dominated by the Culicidae family was the highest (69% of captured insects) compared to the numbers of other orders of insects trapped in the Ambohidray site. This can be explained by the positive phototropism of nocturnal Diptera in relation to the light of the CDC trap. The results showed that the number of individuals in the control and baited traps is different for insects, mosquitoes and *Anopheles* spp. In general, a shift of about 1000 individuals separates the two traps. The numbers of individuals recorded in the baited trap are always greater than those of the control trap. This difference is due to the effects of the products tested on the insects present in the trapping areas.

Six mosquito species (*Cx. decens*, *Cx. tritaeniorhynchus*, *Cx. giganteus*, *An. coustani*, *An. squamosus*, *An. mascarensis*) and the *Anopheles gambiae complex* were captured during this study. This complex consists of eight sibling species of mosquitoes, *An. arabiensis*, *An. bwambae*, *An. melas*, *An. merus*, *An. quadriannulatus*, *An. gambiae s.s*, *An. coluzzii* and *An. amharicus*^22^, that are morphologically indistinguishable^23,24^. Among these species, *An. gambiae s.s* and *An. arabiensis* are known as the principal malaria vectors in the complex^25^. In this experiment, we suppose that these mosquitoes were the dominant species captured in the complex and especially *An. gambiae s.s*, known to have a high degree of anthropophilic behavior^26^, because the traps were placed next to human habitations. In addition, exclusive zoophilic behaviour of *An. arabiensis* reported in Madagascar seems to support this occurs^27^. However, this suggestion is not confirmed by molecular identification which was not conducted. On the other hand, it is reported that *An. arabiensis* (an opportunistic species, predominantly zoophilic) is the species that occur at higher densities among species in the *An. gambiae* complex in several African countries^28,29^.

The presence of *Cx. decens* and *Cx. tritaeniorhynchus* indicates probably that the site is closer to the forest because they are well-known as forest mosquitoes^30^. *An. squamosus* and *An. coustani* are widely present in this site because of the large areas of paddy fields which are favorable to the development of their larvae^31,32^. These species were known as zoophilic species^33^, the presence of livestock, especially zebu in this locality also explains their presence.

**Figure 5:**
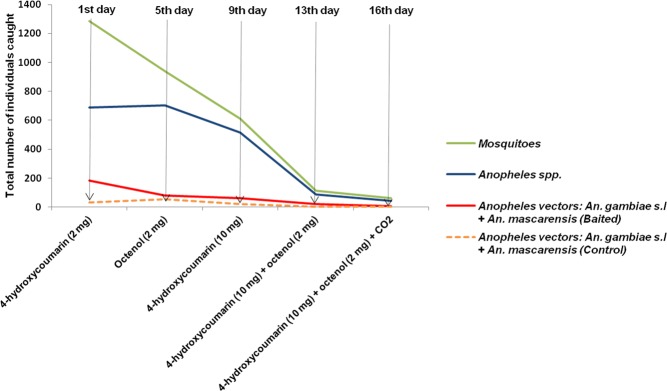
Curve showing the variation in the number of mosquitoes, *Anopheles* spp. and malaria vectors: *An. gambiae s.l* + *An. mascarensis* caught in the baited traps according to the trapping day. Curve in orange shows the number of *An. gambiae s.l* + *An. mascarensis* caught in the control traps.

The number of insects in the traps declines rapidly depending on the progress of the trapping period, indicating the important relation between sampling, population density and trap efficiency. Our results showed that the numbers of insects and mosquitoes trapped in the control and baited traps decrease progressively based on the trapping session. At the beginning of the experiment, we captured 3714 insects and 2406 mosquito individuals for all the traps. This number decreases to 173 insect individuals and 128 mosquito individuals at the end of the experiment. By comparing the number of mosquitoes caught in the baited traps of different products, the results showed that the number of trapped individuals decreases linearly during the 16 days of capture. In the case of *Anopheles* spp., this number remains stable until the fourth day of trapping, and then it gradually decreases. A similar observation was recorded for *An. gambiae s.l*, and *An. mascarensis* together. Thus, a significant decrease in the mosquito population, *Anopheles* spp. and vector species was recorded at the site compared to the number of individuals caught in control particularly for malaria vectors (Fig. 5). These results offer an important research avenue on the possibility of selective eradication of one or more dangerous species in a given area from the use of baited attractant traps. But a longitudinal study should be done to measure their long-term effectiveness.

According to the results, the temperature and relative humidity do not vary too much throughout the experiment. This assumes that these factors did not influence trapping in this study. However, the low number of mosquitoes caught for the last two trapping periods may be due to the decrease in temperature. These results were consistent with the findings of Khan *et al*.^34^ who reported a negative relationship with temperature, relative humidity and *Anopheles* abundance in Jeddah, Saudi Arabia. Bashar and Tuno^35^ reported also not finding any significant correlation between temperatures with mosquito density, but they found a positive association with relative humidity. Other authors found similar findings in their study^36,37^. In contrast, it was reported by Opayele *et al*.^38^ that fluctuations in relative humidity were not significantly correlated with mosquito vectors abundance in Ibadan, Nigeria. Minakawa *et al*.^39^ found also that the influence of temperature on mosquito density was significant for malaria vectors in Kenya. In case of our experiment, we were unable to detect a significant relationship between temperature, relative humidity and mosquito density because there was little variations of these factors during the trapping period. However, longitudinal studies on mosquito trappings through different climatic seasons will be important to observe the influence of these abiotic factors on malaria vectors density in Madagascar.

The results showed that the total number of *Anopheles* spp. recorded in the trap baited with: 4-hydroxycoumarin (10 mg), 4-hydroxycoumarin (10 mg) plus octenol (2 mg) and 4-hydroxycoumarin (10 mg) plus octenol (2 mg) and CO_2_ is higher than that found in the control. This suggests that these products have an attractant effect on *Anopheles* spp. compared to other genus of mosquitoes. The effectiveness of the blend of attractants especially in the presence of CO_2_, a standard attractant, to attract mosquitoes has been reported by other authors^40–43^. Sriwichai *et al*.^44^ demonstrated that CO_2_ significantly attracts the *Anopheles* mosquito by using the CDC light trap. Other studies showed that the use of CO_2_ as bait is not effective in capturing mosquitoes^45,46^.

According to our results, traps with 4-hydroxycoumarin (2 mg) has a high number of malaria vectors, *An. gambiae s.l* and *An. mascarensis* compared to control. But the percentage is low on the order of 25% or only a quarter of all captured *Anopheles spp*. A similar observation was also recorded in the case of the blend 4-hydroxycoumarin (10 mg) and octenol (2 mg). This can be explained by the low population density of these mosquitoes and the high abundance of other *Anopheles* species such as *An. squamosus* and especially *An. coustani* in the study site.

Regarding the efficacy of products with respect to *Anopheles* species, significant attractiveness effect was reported for 4-hydroxycoumarin (2 mg) on *An. gambiae s.l* but also on *An. mascarensis* which is a vector of malaria with anthropophilic behavior^47,48^. However, the average number of captured individuals was quite low in the case of *An. mascarensis* with 6 individuals per trap for four trapping sessions. By increasing the amount of 4-hydroxycoumarin to 10 mg or 5 times compared to the previous one, this attractiveness effect is statistically no more observed towards the two species mentioned above. However, results show that the difference in the average number of malaria vectors in control trap and bait traps is quite significant if the confidence interval is estimated at 90%. In this case, 4-hydroxycoumarin with a mass of 10 mg has also an attractive effect on *An. gambiae s.l*, and *An. mascarensis*. But an increase in the number of repetitions of the tests should be done to have a good reliability of the results.

In this study, octenol at a dose of 10 mg/ml and with a mass deposited of 2 mg did not have a selective attractive effect on *An. gambiae s.l*, and *An. mascarensis*. On the opposite it had a synergistic effect with 4-hydoxycoumarin (10 mg) to attract *An. gambiae s.l* even though the abundance of captured individuals was low. By adding CO_2_ in this blend, the attractiveness effect on *An. gambiae s.l* is no longer observed. This suggests that the addition of CO_2_ in the blend 4-hydroxycoumarin (10 mg) and octenol (2 mg) appears to reduce the selective attractive effect on *An. gambiae s.l*. This is difficult to explain because of the mean number of captured mosquitoes was very low. It would be useful to test this combination of products in the presence of high density of mosquitoes to really observe its effect.

The number of individuals trapped for each species is different depending on the products. It was shown that the number of *An. gambiae s.l*, *An. mascarensis* and *An. squamosus* obtained with 4-hydroxycoumarin (2 mg) was significantly higher compared to the other products that have been tested. This implies that this product at the used dose and quantity has a kairomonal property on these species. Thus, it is important to determine if the product is closer to a human or animal kairomone. In the case of octenol, it presents itself as an animal kairomone because it attracts much more *An. coustani* which has a strong tendency to bite animals especially cattle^49^.

According to the results, the kairomone index for *An. gambiae s.l*, and *An. mascarensis* together in the presence of 4-hydroxycoumarin (2 mg) and 4-hydroxycoumarin (10 mg) plus octenol (2 mg) combination was the most higher (KI = 70%) compared to the kairomone index of the other products. In addition, the kairomone index for each species shown in the results was very high with KI > 65% especially for *An. mascarensis*. This implies that these products have a high selectivity for *An. gambiae s.l*, and *An. mascarensis*. A synergistic effect was observed for the blend 4-hydroxycoumarin (10 mg) and octenol (2 mg) in this experiment. We reported similar results with *Aedes* using the sentinel-type trap^20^. It seems that it is 4-hydroxycoumarin that plays a major role in synergy because even without octenol the selectivity remains high compared to other products. We assume that octenol simply amplifies the attractively effect, but not selective, in this synergism. Thus, it attracts more mosquitoes and any species of *Anopheles* especially *An. coustani* and *An. squamosus*. This reduces its selectivity towards *An. gambiae s.l*, and especially *An. mascarensis* (KI = 0). The results have shown that by increasing the amount of 4-hydroxycoumarin to 10 mg, the kairomone index for *An. gambiae s.l and An. mascarensis* decreases to 48% and 0% respectively, meanwhile those of mosquitoes and *Anopheles* spp. increase significantly. It implies likely that at this quantity, the product may not be optimal to be selective for the malaria vectors and/or that the ratio of malaria vectors in the mosquito population is already much lower than the previous one. According to our results, the kairomone index of blend 4-hydroxycoumarin, octenol and CO_2_ for malaria vectors is about 30%. We can suppose that the CO_2_, a broad-spectrum attractant^50^, is responsible for the decrease of the selectivity for this blend. It is important to note that we discussed about the results of kairomone index of products, regardless of the attractive effect of light trapping.

The CDC light trap allowed for the evaluation of the attractiveness activity of products tested on mosquitoes. However, the difficulty of the attractiveness evaluation using this trap lies in the light because it was reported that the latter has also an attractive effect on bloodsucking insects^51^, especially in regard to the *Anopheles* mosquito. Almost all species belonging to this genus have a nocturnal activity^52,53^. Therefore, the efficacy of the products as attractants is not determined in this study. For this, it is necessary to measure this attractiveness and selective efficacy on mosquito vectors with a much more specific trap without light nor CO_2_, which are known as effective kairomone but without selectivity.

To conclude, this study allowed us to show the attractiveness property of 4-hydroxycoumarin on mosquitoes. 4-hydroxcoumarin at a dose of 10 mg/ml and with a mass deposited of 2 mg generates a significant attractant effect on *Anopheles* spp. and especially on the malaria vector species, *An. gambiae s.l*, and *An. mascarensis* in Madagascar. A significant kairomone selectivity of 4-hydroxycoumarin is also observed on these mosquitoes. In addition, this compound has a synergistic effect with octenol (2 mg) to attract *Anopheles* spp. and also selective on *An. gambiae s.l*, and *An. mascarensis* with kairomone index 70% and 100%, respectively. The selectivity of the products with respect to one or more mosquito species is very important in order to evaluate or even to eliminate them in an area at risk of epidemics. Moreover, in 16 days of experimentations with baited CDC light traps the number of malaria vectors seems to have decreased to almost none and this may be the most important point even if it makes the results analysis more difficult. The use of these selective attracting molecules may be highly ecological in vector control against malaria in Madagascar. However, using 4-hydroxycoumarin at different doses, in areas with high *Anopheles* density where the three main malaria anthropophilic vector species, *An. gambiae s.s*, *An. mascarensis* and *An. funestus* are present, a longitudinal study should be carried out to measure its effectiveness as mosquito control tools and a molecular identification must be performed for the *An. gambiae* complex.

## Methods

### Study site

**Figure 6:**
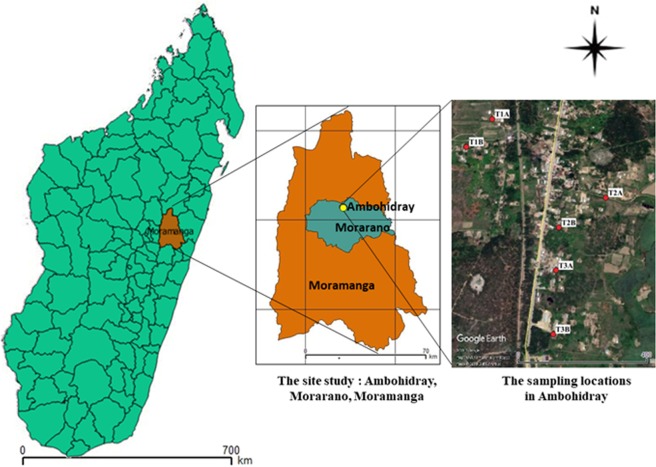
Map showing the study site: Ambohidray and the six locations of the CDC light traps. The maps were generated with software QGIS version 2.18.4 (QGIS Development Team 2017. QGIS Geographic Information System. Open Source Geospatial Foundation Project, http://qgis.osgeo.org). The satellite imagery was obtained from Google (Map data: Google Earth pro, Maxar Technologies, CNES/Airbus; retrieved December 06, 2019).

This study was conducted in the Moramanga district, Morarano commune, Ambohidray village (S 18°36′21.4″; E 048°16′36.4″) (Fig. 6), located in the south-center of the Alaotra Mangoro region 155 km from Antananarivo the capital city of Madagascar. Agriculture and livestock are the main activities of the majority of the villagers of Ambohidray Fokontany. 85% of the populations are rice farmers and rice fields are spread over high surfaces. The composition of mosquito species on the site is not known and the available hosts are mainly human and livestock such as cattle, sheep and poultry. Ambohidray has a warm and temperate climate with an average annual temperature of 19.9 °C and average annual rainfall of 1662 mm.

Sampling was conducted at six locations coded T1A (S 18°36′13.9″; E 048°16′34.8″), T1B (S 18°36′16.7″; E 048°16′32.2″), T2A (S 18°36′21.8″; E 048°16′46.2″), T2B (S 18°36′24.8″; E 048°16′41.5″), T3A (S 18°36′29.1″; E 048°16′41.2″) and T3B (S 18°36′35.5″; E 048°16′40.9″). These locations have been chosen so that the village can be encircled. T1 is at the end of the village, T2 and T3 are respectively located in the center and at the entrance.

### Mosquito trapping

**Figure 7:**
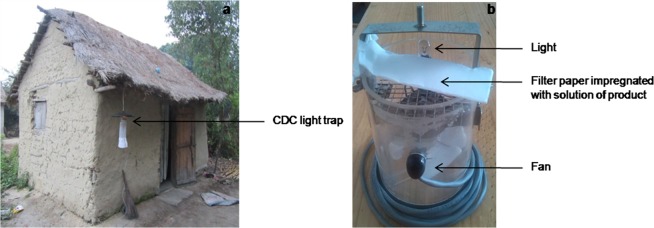
CDC light trap placed next to human habitation (**a**) and filter paper impregnated with a product solution placed next to the lamp of trap (**b**) (Source: Author. Andrianjafy Mbolatiana Tovo).

Mosquitoes were collected during the end of the rainy season in April 2018, using CDC miniature light traps (Model 2836 BQ, 2321 E Gladwick St., Rancho Dominguez, CA 90220 USA) (Fig. 7a). A total of 16 days of trapping was performed in this study and four nights of replication were conducted for each product tested in the six trap locations. All traps were active for 12 hours every night from 6 pm to 6 am. Each trap was powered by a 12 volt battery which was recharged after each trapping session. Six CDC light traps were used, including three control traps containing pure ethanol and three traps baited with an ethanol kairomone solution. Location of control and baited traps is approximately 100 m apart. They were placed close to the human habitation and suspended above the ground at a height of 1.5 m. Control and baited traps were systematically inverted. The attractants were changed for each experiment night. Captured mosquitoes were morphologically identified by using a binocular magnifier (Motic-ST 30) down to the species level by determination keys^54,55^. Identification of other insects remains only at the order level. Temperature and relative humidity data are recorded for each capture session.

### Tested products

Four products were used, two of which were tested alone: 4-hydroxycoumarin (Sigma-Aldrich, Germany) and octenol (CAS 3391-86-4, Aldrich 05284-25 G, Germany) and the two others were tested in combination: 4- hydroxycoumarin plus octenol and 4-hydroxycoumarin plus octenol plus carbon dioxide (CO_2_) (Table 2). CO_2_ was produced locally in 1.5 liter plastic bottles containing 10 g of yeast, 40 g of brown sugar and 500 ml of warm water^56^. A rubber hose connects directly the CO_2_ source and the CDC light trap.

**Table 2:** Products tested with the doses and masses used in the study.

Products	Mass deposited (mg)	Dose (mg/ml)
4-hydroxycoumarin	2 10	10
Octenol	2	10
4-hydroxycoumarin + octenol	10/2	10/10
4-hydroxycoumarin + octenol + CO_2_	10/2	10/10

A concentration of 10 mg/ml was prepared for each product. For 4-hydroxycoumarin and octenol, a volume of 200 μl of an ethanolic solution of products, equivalent to a mass of 2 mg, was deposited on a strip of filter paper (4 × 20 cm, VWR, France) by using a micropipette. A volume of 1000 μl of an ethanolic solution of products with the same concentration, equivalent to a mass of 10 mg, was also deposited for 4-hydroxycoumarin. For the blends, a concentration of 10 mg/ml was also prepared for each product, but the deposited mass was different for 4-hydroxycoumarin and octenol with 10 mg and 2 mg, respectively. The paper impregnated with solution of product was then attached to a support just above the fan, next to the lamp of the trap (Fig. 7b).

### Data analysis

The attractiveness activity of products was measured by comparing the average number of mosquito individuals caught in control and baited traps by using the student *t-test*. The average number of mosquitoes and *Anopheles* spp. in the baited traps of the different products were compared using *ANOVA test* and *LSD* multiple comparison was used to sort the differences between them.

To evaluate the selectivity of the products, the kairomone index (KI) was calculated. KI corresponds to the ratio of the difference of the total number of mosquitoes or *Anopheles* spp. or malaria vectors in baited and control traps by the sum of the total number of mosquitoes or *Anopheles* spp. or malaria vectors in baited and control traps.

$${\rm{KI}}\,{\rm{mosquitoes}}=\frac{{\rm{Total}}\,{\rm{number}}\,{\rm{of}}({\rm{mosquitoes}}\,{\rm{in}}\,{\rm{baited}}\,{\rm{trap}}-{\rm{mosquitoes}}\,{\rm{in}}\,{\rm{control}}\,{\rm{trap}})}{{\rm{Total}}\,{\rm{number}}\,{\rm{of}}({\rm{mosquitoes}}\,{\rm{in}}\,{\rm{baited}}\,{\rm{trap}}+{\rm{mosquitoes}}\,{\rm{in}}\,{\rm{control}}\,{\rm{trap}})}$$

All data were analyzed using the 2018 XLSTAT Software. The confidence interval was estimated at 95%.

## Data Availability

The datasets generated during the current study are available from the corresponding author on reasonable request.
